# Any Role of PIK3CA and PTEN Biomarkers in the Prognosis in Oral Squamous Cell Carcinoma?

**DOI:** 10.3390/life10120325

**Published:** 2020-12-03

**Authors:** Anna Starzyńska, Paulina Adamska, Aleksandra Sejda, Monika Sakowicz-Burkiewicz, Łukasz Jan Adamski, Giulia Marvaso, Piotr Wychowański, Barbara Alicja Jereczek-Fossa

**Affiliations:** 1 Department of Oral Surgery, Medical University of Gdańsk, 7 Dębinki Street, 80-211 Gdańsk, Poland; paulina.adamska@gumed.edu.pl (P.A.); lukasz.adamski@gumed.edu.pl (Ł.J.A.); 2 Department of Pathomorphology, University of Warmia and Mazury, 18 Żołnierska Street, 10-561 Olsztyn, Poland; aleksandra.sejda@uwm.edu.pl; 3 Department of Molecular Medicine, Medical University of Gdańsk, 17 Smoluchowskiego Street, 80-214 Gdańsk, Poland; ssak@gumed.edu.pl; 4 Division of Radiotherapy, IEO European Institute of Oncology, IRCCS, 435 Ripamonti Street, 20-141 Milan, Italy; giulia.marvaso@ieo.it (G.M.); barbara.jereczek@ieo.it (B.A.J.-F.); 5 Department of Oncology and Hemato-Oncology, University of Milan, 7 Festa del Perdono Street, 20-112 Milan, Italy; 6 Department of Oral Surgery, Medical University of Warsaw, 6 Binieckiego Street, 02-097 Warsaw, Poland; piotrwychowanski@wychowanski.pl

**Keywords:** PIK3CA amplification, PTEN loss, phosphatidylinositol 3-kinase pathway, oral squamous cell carcinoma

## Abstract

Oral squamous cell carcinoma (OSCC) accounts for 95% of the lesions in the oral cavity. Despite development in OSCC management, the outcome is still unsatisfactory. Identification of new therapies in OSCC is urgently needed. One objective of such treatment may be a signaling pathway of phosphatidylinositol 3-kinase. The study group included 92 patients treated for OSCC at the University Clinical Centre in Gdańsk, Poland. Study was performed on formalin-fixed paraffin-embedded samples from primary OSCC. Phosphatidylinositol-4,5-bisphosphate 3-kinase (PIK3CA) and phosphatase and tensin homolog encoded on chromosome 10 (PTEN) protein expression was assessed by immunohistochemistry (IHC). *PIK3CA* gene copy number was analyzed using chromogenic and silver in situ hybridization where molecular probes are marked by chromogens and silver ions. PIK3CA IHC H-score ≥ 70 was found in 51.65% patients, and loss of PTEN protein was noticed in 31.46% cases. PIK3CA amplification was detected in 5 tumors. In the case of PTEN protein expression, there was an inverse correlation with the T stage of the primary tumor (*r =* −0.243) and positive correlation with a 5-year survival (*r =* 0.235). The number of copies of the *PIK3CA* gene was associated with the tumor grading (*r =* 0.208). The present study shows that loss of PTEN protein and the grading (*p =* 0.040), distant metastases (*p =* 0.033), smoking (*p =* 0.016), and alcohol abuse (*p =* 0.042) were prognostic factors for the survival of patients with OSCC. In contrast, the presence of amplification and OSCC on the floor of the mouth resulted in a nearly six-fold increase in the risk of shortening survival (*p =* 0.037). Our finding suggests a potential prognostic significance of PTEN loss and PIK3CA amplification in OSCC. Future studies are needed to confirm our results.

## 1. Introduction

Oral squamous cell carcinoma (OSCC) is the most common malignant tumor in oral cavity. It accounts for 95% of the malignant lesions in this location with the incidence rate about 300,000 worldwide [1,2]. The mortality rate had not changed for many decades. OSCC is now responsible for 5% of all cancer-related deaths [3,4].

The lesion mainly affects men over 50 years of age [5]. The major risk factors for oral cancer development are tobacco, betel quid, and alcohol [6]. The frequency of tobacco smoking (cigarettes per day) and the duration of tobacco smoking (years) increased the OSCC risk incidence (dose-response relationships) [6]. Alcohol alone is found to increase risk of oral carcinoma only when consumed at high doses (> 15 units per week) [7]. In recent years, a rapid increase in OSCC cases in people under 45 years of age has been observed. Most of these cases were associated with Human Papillomavirus infection (HPV) [5,8,9,10].

OSCC can be located on the anterior two-thirds of the tongue, floor of the mouth, buccal mucosa, gums, and hard palate [1,2]. The most common symptoms are redness of mucous membrane, ulceration, pain, and Vincent’s symptom. In more severe cases, trismus, dysphagia, or odynophagia may be present [8,11,12].

At the time of diagnosis, every tenth patient presents with distant metastases or dissemination to the local lymph nodes [11,12]. Tumor size, depth of invasion, distant metastases, and positive margins after surgery remain the most important prognostic factors [13].

Prognostic marker research is a growing area in the field of oncology. The most common molecular prognostic factors found in OSCC are tumor protein P53 (P53), epidermal growth factor receptor (EGFR), cMET (tyrosine-protein kinase Met pathway), transforming growth factor beta/SMAD protein pathway (TGFβ/SMAD pathway), RAS protein/mitogen-activated protein kinases pathway (RAS and MAPK pathway), or NOTCH protein pathway (NOTCH) [14,15,16,17,18].

The intracellular phosphatidylinositol 3-kinase signaling pathway (PI3K) is involved in the number of processes connected with cancer development and progression. Among others it has an impact on cell differentiation, migration, growth, proliferation, regulation of gene expression, cellular metabolism, cytoskeletal re-assortment, protein transport, and actin polymerization [19,20,21,22].

Activation of PI3K pathway is mainly regulated by receptor tyrosine kinases (RTK). After ligand binding (growth factors, cytokines, hormones), the receptor undergoes autophosphorylation which results in PI3K transposition into cell membrane by the receptor phosphotyrosine residues. This leads to stimulation of the p85 regulatory subunit of PI3K, which further activates the catalytic p110 domain (PIK3CA, phosphatidylinositol-4,5-bisphosphate 3-kinase) [22,23] responsible for the phosphorylation of phosphatidylinositol 4,5-bisphosphate (PIP2) to phosphatidylinositol 3,4,5-trisphosphate (PIP3). PIP3, with the use of pleckstrin homology (PH) domains, recruits signaling proteins, such as AKT (serine-threonine kinase), near the cytoplasmic membrane, where AKT is activated by PDK1 (3-phosphoinositide-dependent kinase-1) [19,20,21,22]. AKT allows oncogenic transformation of cells by stimulation of a number of proto-oncogenes and suppression of tumor suppressor genes, resulting in cell proliferation and inhibition of apoptosis [18]. Downregulation of the PI3K pathway is a result of PIP3 dephosphorylation by phosphatase and tensin homolog encoded on chromosome 10 (PTEN) protein (phosphatase and tensin homolog encoded on chromosome 10) [24,25,26]. Loss of *PTEN* gene expression results in increased AKT activity and unstopped cell proliferation [27,28,29].

The p110 subunit of PI3K is encoded by the *PIK3CA* gene located on 3q26.32 locus. Gene activation in head and neck, lung, breast, colon, ovary, or cervix cancers may lead to neoplastic transformation, usually as a result of H1047R (exon 20), E542K, and E545K (exon 9) point mutations or gene amplifications [23,30,31,32,33,34]. As for the *PTEN* gene, its inactivation by deletions and missense point mutation frequently observed in prostate, breast, lung, endometrial and colorectal cancer is responsible for unstable cell proliferation [23,27,29,35,36,37]. Many studies of aberrations and mutations in PI3K in other tumors were described, but the literature documenting *PIK3CA* and *PTEN* genetic aberrations in OSCCs is limited [23,27,29,30,31,32,33,34,35,36,37]. There are few studies concerning PIK3CA amplification and PTEN loss, with indistinctive results.

The following prognostic factors were described for OSCC: cyclin dependent kinases (CDKs), survivin, minichromosome maintenance (MCM) proteins, multidomain protein kinases BUBR1 (BUBR1), heat shock proteins (HSPs), urokinase plasminogen activator receptor (UPAR), and CD44 antigen (CD44). Survival rate was lower in patients with CDK1 positive expression when compared with CDK1 negative cases. High expression of survivin, MCM, and BUBR1 positive expression was correlated with shorter survival. In contrast, HSP27 and CD44 protein expression was associated with better overall survival [38]. *PIK3CA* point gene mutations were identified in 0% to 13.92% of patients, according to different studies, and *PTEN* deletion was found in 5% of cases. *PIK3CA* gene amplification has been described in 9% to 50% of OSCC patients [39]. Current studies on various malignancies focus on molecular biomarkers. Targeted therapy for OSCC and head and neck squamous cell carcinoma (HNSCC) are still a new concept. Up to date, some studies have shown that inhibitors of PI3K/AKT/PTEN pathway can support treatment in recurrent and metastatic oral or head and neck cancers [40,41,42,43]. Use of temsirolimus in combination with cetuximab or carboplatin and paclitaxel in head and neck cancer had been studied. In HNSCC, everolimus was combined with cisplatin and docetaxel as induction therapy for radiation. In this clinical trial, the 2-year overall survival rate was 91%, and the progression-free survival rate at 2 years was 76.6% in patients pretreated with everolimus [44]. In another study, everolimus was used together with cisplatin and concurrent radiation, resulting in the 2-year overall survival rate of 92% and the 2-year progression-free survival rate of 85% [45]. The best response has come from everolimus in combination with carboplatin and paclitaxel as induction therapy in patients with unresectable, locally advanced HNSCC. The presented results showed 2.6% complete response, 76.3% partial response, and 21% stable disease in the 38 patients [46]. The PI3K-Akt signaling pathway might be an interesting therapeutic approach.

In our study, we evaluated PIK3CA and PTEN protein expression and gene copy number of *PIK3CA* gene in patients with OSCC in relation to clinical characteristics and survival. The mutual correlations and the prognostic significance of biomarkers were assessed.

## 2. Results

### 2.1. Characteristic of Patients

Most of the patients in the analyzed group were men (67.39%). The average age was 61.52 years, ranging from 36 to 89. The majority of the tumors were located in the floor of the mouth (31.52%) and in the tongue (19.67%). According to the degree of histopathological differentiation, the most frequently reported tumors were assessed as grade 2 (46.74%), then grade 1 (41.30%). The most numerous group included patients with T2 (26.09%) and T4 (20.65%) cancer stages. Lymph nodes were negative (N0) in 40.22% of patients. None of the patients had distant metastases at the time of diagnosis. In our patients, OSCC was diagnosed mainly in stage IV (30.43%). Nicotinism and alcoholism were declared by 53.26% and 15.21% of respondents, respectively. Alcoholism was reported mainly by men (*p =* 0.011). Mean pack-years smoking history was 20.71.

Surgery was the most common treatment method. Almost 35% of patients underwent radical OSCC removal. In these patients, it was the only method of treatment. Patients over 60 years of age statistically more often underwent surgical treatment (*p =* 0.029).

The mean follow-up period was 3.74 years, ranging from 0.08 to 9.83 years. Over 67% of patients did not survive 5 years. There was a large decrease in survival within the first 16 months of cancer treatment. The study analyzed overall survival (OS) based on the clinical and pathological data using Cox proportional risk regression. Detailed clinicopathological data are presented in Table 1.

### 2.2. Immunohistochemistry Assessment of PIK3CA and PTEN Protein

#### 2.2.1. PIK3CA Protein

The obtained H-score (hybrid scoring system method) ranged from 0 to 200 with the average of 62.31 and the median of 70. The number of patients with positive expression was 54 (59.34%), and the number of those with high immunostaining level with H-score cut-off, at 70, was 47 (51.65%). None of the patients had a maximum H-score (300). There was no significant correlation between PIK3CA protein expression evaluated as a continuous variable and clinico-pathological factors (Table 2). Similarly, no significant differences were between the groups with high- and low-level expression of PIK3CA (Table 1).

**Figure 1:**
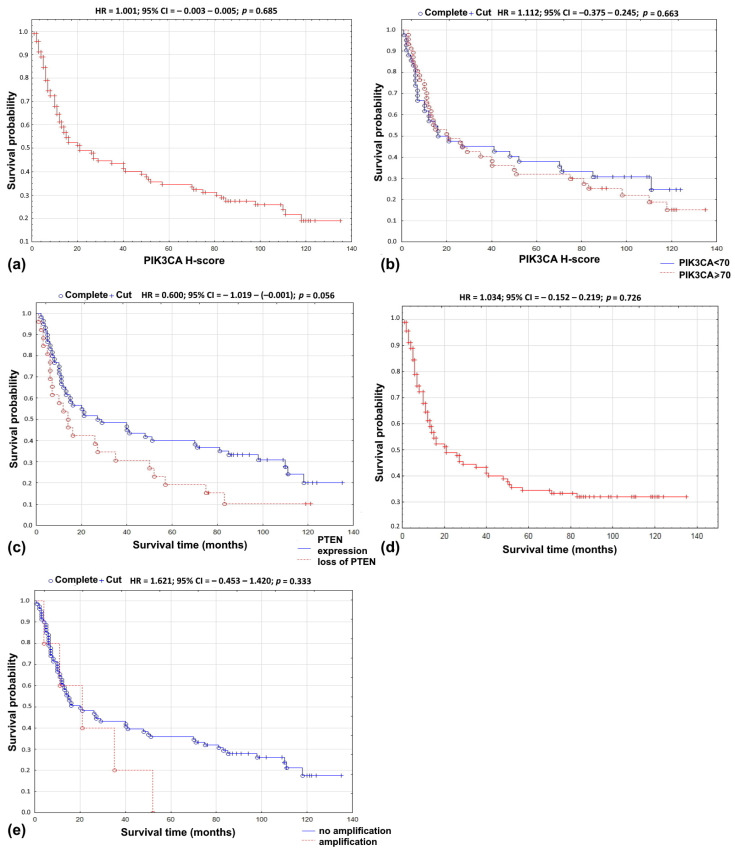
Overall survival probability curve according to: (**a**) PIK3CA protein expression as continuous variable; (**b**) PIK3CA—H-score using 70 as cut-off point; (**c**) loss of PTEN; (**d**) *PIK3CA* copy number as continuous variable; (**e**) *PIK3CA* amplification.

In univariate Cox’s analysis, PIK3CA protein expression treated as a continuous variable was not related to overall survival (HR = 1.001; 95% CI = −0.003–0.005; *p =* 0.685; Figure 1a). Similarly, no impact on survival was found when PIK3CA protein expression was divided into two groups, with high and low protein expression (HR = 1.112; 95% CI = −0.373–0.245; *p =* 0.663; Figure 1b). No association was found using the multivariate Cox model adjusting for age, gender, tumor localization, grading, staging, treatment modality, recurrence, tobacco smoking, or alcohol abuse (Table 3).

#### 2.2.2. PTEN Protein

A loss of PTEN protein expression was shown in 31.46% of patients. An inverse correlation between PTEN protein expression has been demonstrated with T stage (*r =* −0.243; *p =* 0.043) and 5-year survival rate (*r =* 0.2350; *p =* 0.028; Table 2). Loss of PTEN was more common in T3 and T4 tumors compared to T1 and T2. Eighty-two percent of patients with loss of PTEN did not survive 5 years. An increasing trend in the frequency of PTEN protein loss has been observed in men (*p =* 0.171) with metastases to regional lymph nodes (*p =* 0.263), in stage IV (*p =* 0.250), and in patients with positive smoking status (*p =* 0.055) (Table 1).

Loss of PTEN resulted in lower 5-year survival rate (*r =* 0.2350; *p =* 0.028; Table 2). In univariate analysis, the loss of PTEN protein did not influence overall survival (HR = 0.600; 95% CI = −1.019–−0.001); *p =* 0.056; Figure 1c). In the multivariate Cox’s regression analysis, the loss of PTEN protein and grading (*p =* 0.040), distant metastases (*p =* 0.033), smoking (*p =* 0.016), and alcoholism (*p =* 0.042) were associated with shorter overall survival time (Table 3).

### 2.3. Analysis of the PIK3CA Gene Copy Number Using Chromogenic and Silver In Situ Hybridization (CISH/SISH)

The average value of the *PIK3CA* gene copy number was 2.52 (standard deviation 1.26, median 2.5). The average value of the *PIK3CA copy number/chromosome 3 cytochrome* ratio (*PIK3CA/CEP3* ratio) was 1.10; standard deviation was 0.55, and median was 1.14. Amplification was demonstrated in 5.62% of patients.

*PIK3CA* gene copy number tended to be associated with higher grading (*r =* 0.208; *p =* 0.050; Table 2). It has been demonstrated that *PIK3CA* gene amplification was more common in tumors located in the lower gingiva compared to other locations (*p =* 0.040). No differences were found in relation to other data (Table 1).

In univariate analysis, no relationship was found between the *PIK3CA* gene copy number and overall survival (HR = 1.034; 95% CI = −0.152–0.219; *p =* 0.726; Figure 1d). Similar results were obtained in the analysis of the *PIK3CA/CEP3* ratio (HR = 1.207; 95% CI = −0.232–0.608; *p* = 0.392) and the gene amplification (HR = 1.621; 95% CI = −0.453–1.420; *p =* 0.333; Figure 1e). In the multivariate Cox proportional risk regression model, the *PIK3CA* gene copy number and the *PIK3CA/CEP3* ratio were not associated with survival, age, gender, tumor localization, grading, staging, smoking, or alcohol abuse. In contrast, the presence of amplification and location of the tumor in the lower gingiva resulted in an almost six-fold increase in the risk of shortening the survival rate (HR = 5.783; 95% CI = 0.054–3.456; *p =* 0.037) (Table 3).

### 2.4. Combined PIK3CA Protein Level and PIK3CA Gene Copy Number Analysis

For 96.74% (*n* = 89/92) samples, results were obtained regarding the PIK3CA protein level and the *PIK3CA* gene copy number. In 60% (*n* = 3/5) of patients with *PIK3CA* gene amplification, PIK3CA protein expression was observed.

The patients whose samples presented with amplification belonged to the group with high PIK3CA protein expression. There was no relationship between *PIK3CA* gene copy number and PIK3CA protein expression analyzed as a continuous variable (*r =* 0.058; *p =* 0.587) or when divided into high and low protein expression groups (*r =* 0.025; *p =* 0.819). Similar results were obtained for the analysis of the *PIK3CA/CEP3* ratio, regardless of whether it was analyzed as a continuous feature (*r =* −0.011; *p =* 0.917) or in correlation with low and high expression PIK3CA level (*r =* −0.018; *p =* 0.864). *PIK3CA* gene amplification did not correlate with PIK3CA protein expression (*r =* 0.048; *p =* 0.605).

### 2.5. Combined PIK3CA and PTEN Protein Expression Evaluation

For 95.65% of patients, the results were available regarding the immunohistochemical reaction of both PIK3CA and PTEN proteins. In 15.91% of patients (*n* = 14/88) with loss of PTEN protein, PIK3CA protein expression was not found in the tumor cells. In 13.64% (*n* = 12/88) of samples, no expression of PTEN occurred with a positive PIK3CA color reaction.

There was no relationship between the loss of PTEN protein and status of the PIK3CA protein analyzed as a continuous variable (*r =* 0.036; *p =* 0.605) nor PIK3CA divided into high and low protein expression (*r =* 0.038; *p =* 0.713).

### 2.6. Combined Loss of PTEN Protein and PIK3CA Gene Copy Number Evaluation

For 93.78% (n = 86/92) of patients, results were obtained regarding the expression of PTEN protein and the *PIK3CA* gene copies. In 40.00% (n = 2/5) of patients who had *PIK3CA* gene amplification, loss of PTEN protein expression was observed.

There was no relationship between PTEN protein loss and *PIK3CA* gene copy number when treated as continuous variable (*r =* 0.016; *p =* 0.885), *PIK3CA/CEP3* ratio (*r =* 0.045; *p =* 0.681), or gene amplification (*r =* −0.053; *p =* 0.629).

## 3. Discussion

Our study demonstrated that in oral squamous cell carcinoma PIK3CA protein is frequently expressed (59.34% patients). In other studies, PIK3CA protein expression was observed in 38.90–100% cases [47,48,49,50]. The use of different cell staining scales can severely affect the degree of positive results. The simplest one categorizes staining as 0 (no staining), + (weak), ++ (moderate) and +++ (strong) based only on the intensity of staining [26,49]. In another study, more complex staining evaluation was performed, where samples with less than 10% of positively stained cells were defined as negative results, those from 10% to < 30% as weakly positive, from ≥ 30% to < 50% as moderately positive, and above ≥ 50% of positive cells as highly positive [47]. A modified semi-quantitative scoring system similar to ours was used by Won HS et al. In that study, an immunoreactive score (IRS) based on the multiplication between positive cells proportion score (0–4) and staining intensity score (0–3) was determined. IRS > 6 was consistent with positive PIK3CA protein expression [48].

There is no single best technique to determine the degree of immunohistochemical staining preparations. The H-score seems to be the most popular one. In oral squamous cell carcinoma, this method was used for the CD44, CD73, and mediator of DNA damage checkpoint protein 1 (MDC1 protein) evaluation [51,52,53]. For PIK3CA, immunohistochemistry (IHC) detection in other solid lesions, the cut-off value describing positive samples was individually determined, e.g., in non-small cell lung cancer and colorectal cancer, the cut-off point was H-score ≥ 10 [31,54]; in stomach cancer it was for over 20% of stained cells [55]. H-score of 10 is widely accepted for assessing the level of proteins, e.g., in studies of epidermal growth factor receptor (EGFR). We used the H-score of 10 as a cut-off point to evaluate expressions as positive. We divided the patients by the median and as a result, 2 groups with either low or high expression were distinguished. The assessment methods should be harmonized to make the results reliable and comparable [56,57].

In our study, there were no statistically significant correlations between the high PIK3CA protein level and clinicopathological characteristics, which is consistent with most OSCC studies conducted so far [46,47,50]. Garg R et al. showed that, in the patients with more advanced disease, the intensity of staining is stronger [49]. The status of the PIK3CA protein level was also examined in other solid tumors [46,54,58,59,60,61,62,63]. In head and neck squamous cell carcinoma, it has been shown that the increase in the status of the PIK3CA protein was associated with the progression of lesions from dysplasia to invasive cancer [58]. PIK3CA protein expression was correlated with stage II-IV disease and poor differentiation in non-small cell lung cancer [54]. In esophageal and gastric carcinoma, the presence of node metastases was significantly correlated with positive staining for PIK3CA [55,60]. PIK3CA protein expression was correlated with *PIK3CA* gene amplification in the lung [54] and colorectal cancer [62].

In our study, no relationship was found between the PIK3CA protein expression and patient survival in univariate and multivariate analysis. Among the available studies, only one studied the effect of protein expression on survival. Won SH, et al. showed that the PIK3CA was not an independent predictor for relapse-free survival [48]. In ovarian, breast, and head and neck cancer, a statistically significant relationship between longer overall survival and PIK3CA positive color reaction was reported [61,64,65].

In this study, 31.46% of cases demonstrated loss of PTEN protein. In other studies on OSCC and loss of PTEN, no protein expression was observed in 1.70% to 63.01% of patients [48,50,66,67,68,69,70,71]. The wide range of results is due to different methods of staining evaluation. We found that loss of PTEN protein was associated with a higher T stage. Moreover, we observed a statistically irrelevant tendency to the loss of PTEN expression occurrence in men, well- and moderately-differentiated tumors, primary T4 tumor, metastases to regional lymph nodes, and stage IV cancers. In other studies, the loss of PTEN was correlated with age, grading, stage, the occurrence of distant metastases and relapses in patients with OSCC [66,67,69,72]. In solid tumors, loss of PTEN was frequently observed. On average, in 56% of patients with squamous cell carcinoma of the larynx, PTEN was negative, as well as in 39.13% of non-small cell lung cancer, 56.74% of gastric cancer, 34.88% of colorectal cancer, and in 38.10% of breast cancer patients [73,74,75,76,77]. In non-small cell lung cancer, similarly to our findings, loss of PTEN protein was correlated with the T stage of primary lesion. Additionally, correlations were found with grading, lymph node, and distant metastases [74]. Opposite to our results, in larynx loss of PTEN protein expression was found in poorly-differentiated tumors [73].

We found no relationship between loss of PTEN expression and patient survival in univariate analysis. A large decrease in survival within the first 16 months of cancer treatment was observed. This may indicate that this is the most dangerous period of treatment. In contrast, multivariate analysis showed the effect of loss of PTEN protein and the grade, distant metastases, tobacco smoking, and alcoholism on overall survival. Only two studies showed that the loss of PTEN expression was associated with overall survival in OSCC [66,78]. In non-small cell lung cancer and stomach cancer, statistically significant correlations were demonstrated between longer survival time and negative PTEN color reaction [74,75].

The presence of *PIK3CA* gene amplification was demonstrated in 5.62% of our patients. The size of the group was not large; therefore, these are preliminary results. In existing oral cancer studies, an increase in the frequency of *PIK3CA* gene amplification has been reported in 16.70% to 100% of patients [79,80,81,82,83,84,85]. The variation in the frequency of amplification may be due to the sample size (studies ranged from 7 to 220 patients), method used to analyses the gene copy, or other factors like ethnic group/race of patients participating in the study (Caucasian or Mongoloid race). In the study by Sticht et al., overall frequency of *PIK3CA* copy numbers evaluated by FISH was 39.0% [84]. Analysis of the gene copy number using PCR methods identified amplification in > 50% of primary OSCC samples [81].

To our knowledge, the chromogenic in situ hybridization/silver in situ hybridization (CISH/SISH) technique has not yet been used to analyze the copy number of the *PIK3CA* gene in OSCC cells. Other methods, such as FISH (fluorescent in situ hybridization), PCR (polymerase chain reaction), RT-PCR (reverse transcription polymerase chain reaction), aCGH (array comparative genomic hybridization), and MLPA (multiplex ligation-dependent probe amplification), were more commonly used [80,81,82,83,84,85]. The most popular method for gene copy number evaluation is FISH. However, there are some advantages of the CISH/SISH method over FISH, such as full process automation, and thus full standardization of the results, probes examined using a standard bright-field microscope, so cell morphology and tissue architecture can be seen, lower costs, and long-term storage of preparations without the risk of loss of signals [79,80,81,82,83,84].

We showed the relationship between the number of copies of the *PIK3CA* gene and the grade of the tumor. *PIK3CA* gene amplification was more common in tumors located in the lower gingiva compared to other locations. In other studies, the *PIK3CA* gene copy number was associated with grade, stage, recurrence, and relapse in smokers [80,81,84]. Amplification was correlated with the stage of cancer (stage III/IV vs. I/II) and metastases to local lymph nodes [86]. An increase in the copy *PIK3CA* gene number evaluated by CISH and SISH methods was also noted in lung [87,88], uterine and ovarian cancer [88], and gastric [89].

In this study, no relationship was found between the increase in *PIK3CA* gene copy number, the *PIK3CA/CEP3* ratio, nor the occurrence of *PIK3CA* gene amplification and patient overall survival in univariate analysis. In the multivariate Cox proportional risk regression model, the copy number of the *PIK3CA* gene, the *PIK3CA/CEP3* ratio, and clinical-pathological factors were not prognostic factors for survival. In contrast, the presence of amplification and tumor location in the lower gingiva resulted in an almost six-fold increase in the risk of shortening overall survival. In other studies, the results were similar [82,84]. No relationship between *PIK3CA* gene amplification and overall survival or disease-free survival were found [82,84]. Prognostic value of *PIK3CA* mutation has been observed for a number of tumors, particularly for breast cancer. For example, the identification of the *PIK3CA* gene mutation makes it possible to identify a special group of patients (*PIK3CA*-positive breast cancer) who receive the greatest benefit from molecular-targeted anti-PI3K therapy [90,91,92].

In this study, no relationship was found between the level of PIK3CA and the number of copies of the *PIK3CA* gene, the *PIK3CA/CEP3* ratio or gene amplification. In other studies on oral squamous cell carcinoma, results were similar [80]. On the other hand, PIK3CA protein expression was correlated with *PIK3CA* gene amplification in lung and colorectal cancer [54,62].

Tissue microarrays were used instead of the immunohistochemistry procedure on standard paraffin block slices. As a result, the cost of the study was reduced, more samples were under the same reaction conditions and were subjected to IHC testing, and the original paraffin block could be used for subsequent tests (lower consumption of tissue material). Since the material was obtained from two very small areas in the tumor and the oral squamous cell carcinoma exhibits heterogeneity, this method is limited. Protein expressions and genetic aberrations might be different in other parts of tumor [91].

## 4. Materials and Methods

### 4.1. Materials

The retrospective study included 92 patients with OSCC, treated at the University Clinical Centre in Gdańsk, Poland, between 2007−2012. The protocol of the study was approved by the local bioethics committee at the Medical University of Gdańsk (NKBBN/60/2016). During the research, the anonymity of the patients was preserved.

Clinical data and treatment methods were obtained on the basis of available medical documentation. Patients with local recurrence, other malignant tumor, or incomplete medical records were excluded from the study. Study was performed on formalin-fixed paraffin-embedded samples from primary OSCC. The samples were obtained from tumor resection or from biopsy specimens. Socio-pathological data, such as age, gender, addictions, cancer localization, grading, staging, and survival, were analyzed. The 8th edition of the T—primary tumor/regional lymph nodes—N, distant metastasis—M (TNM) classification was used to assess the staging. Hematoxylin and eosin slides were re-evaluated to confirm squamous carcinoma diagnosis and pathological features (e.g., grade). During the research, the patients were fully anonymous.

### 4.2. Tissue Microarray

Tissue microarrays (TMA) were prepared using Manual Tissue Arrayer MTA 1 (Beecher Instruments Inc, Sun Prairie, WI, USA). Two representative cores, both of 0.4 mm in diameter, were taken from each paraffin block and placed in the secondary paraffin block. The secondary blocks were cut in the microtome sections of 4 μm in diameter and placed on a microscope slide. The TMAs were submitted to the immunohistochemical, chromogenic, and silver in situ hybridization procedure.

### 4.3. Immunohistochemistry

**Figure 2:**
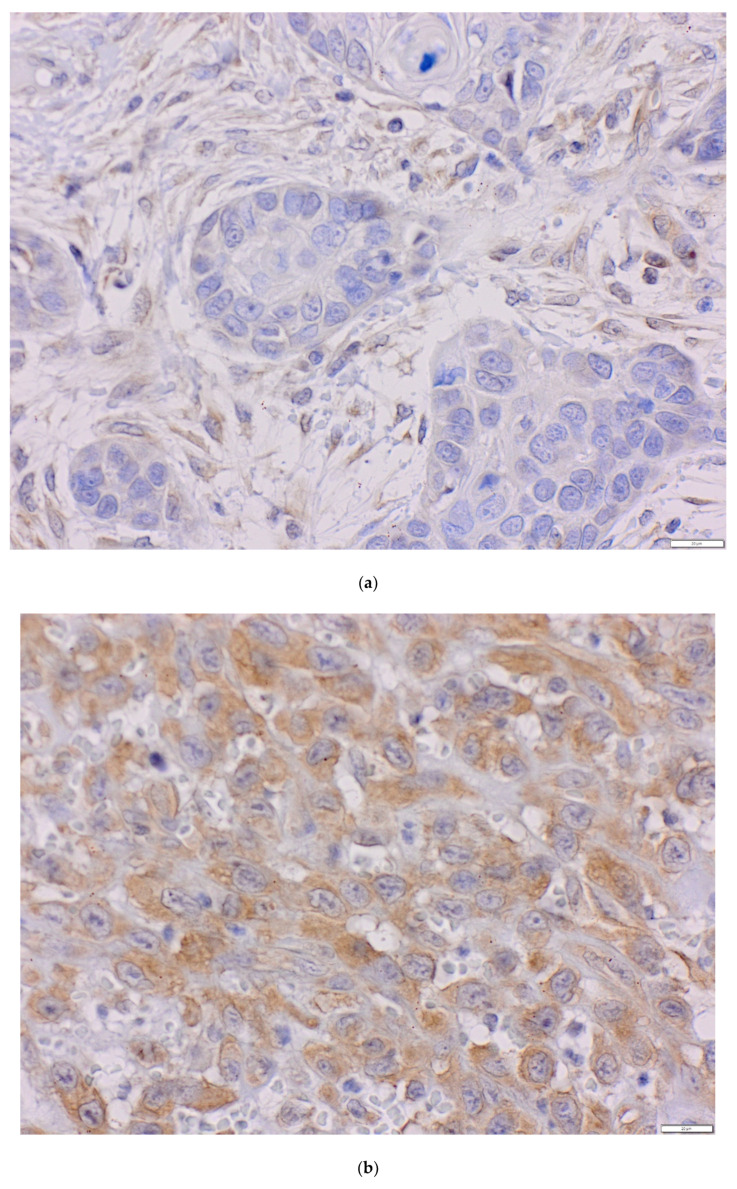

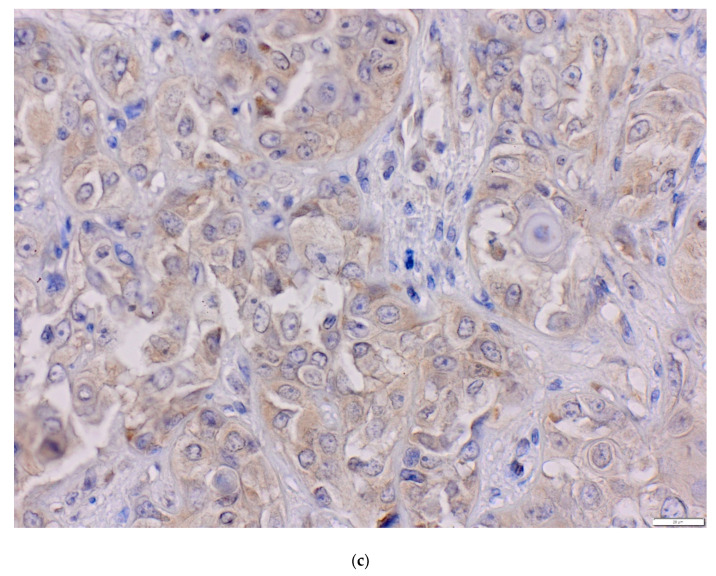
The level of PIK3CA protein in oral squamous cell carcinoma (OSCC) (magnification ×40) (**a**) H-score = 0, (**b**) H-score = 70, (**c**) H-score = 200.

**Figure 3:**
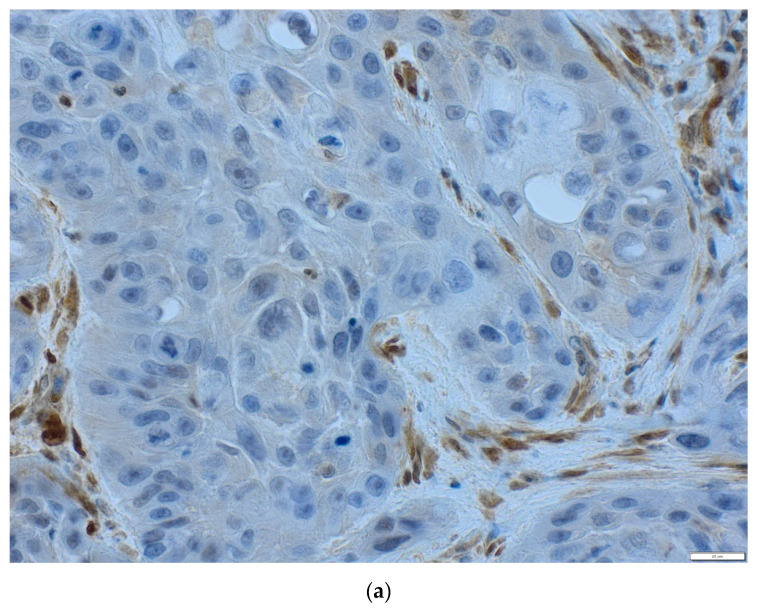

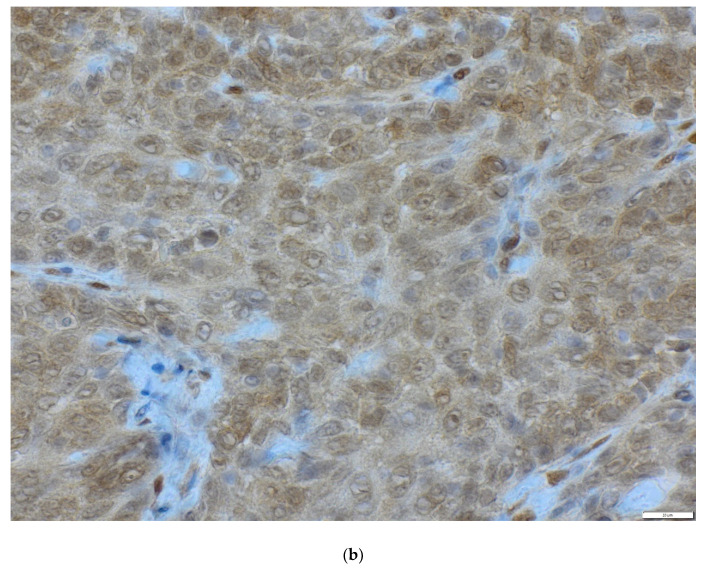
The status of PTEN protein in OSCC (magnification ×40) (**a**) loss of PTEN, (**b**) positive PTEN expression.

Immunohistochemistry procedure was performed using the Ventana G11 system (CONFIRM™, Ventana Medical Systems, Tucson, AZ, USA). PIK3CA and PTEN protein expressions were assessed using the rabbit anti-human monoclonal antibody: PI3 Kinase p110α (C73F8) and PTEN (D4.3) (Cell Signaling, Danvers, MA, United States). Primary antibodies were used at 1:50 dilutions. Mucosa of the colon was the positive control. IHC procedure was conducted following the manufacturer’s instructions. Expression of PIK3CA protein was analyzed using the H-score (hybrid scoring system) method, which is the sum of the products of staining intensity (0–3) and the percentage of cells in each intensity zone (0–100%). The H-score ranged from 0 to 300. For each patient, results from two cores, or less, if depleted, were averaged and compared with the core with the highest score. Samples with H-scores ≥10 were considered positive expressions. PIK3CA was assessed as a continuous variable and divided into two groups: low and high PIK3CA protein expression with a cut-off point of 70—the median for H-score (Figure 2a–c) [51,52,53]. PIK3CA protein expression was evaluated for 91 probes. One patient was not included due to TMA core loss. The result of PTEN protein expression was analyzed in a binomial system. The value of 0 was consistent with the loss of PTEN protein nuclear expression, and the value of 1 indicated the presence of PTEN protein (Figure 3a,b). Three patients were not included due to TMA core loss.

### 4.4. Chromogenic and Silver In Situ Hybridization

**Figure 4:**
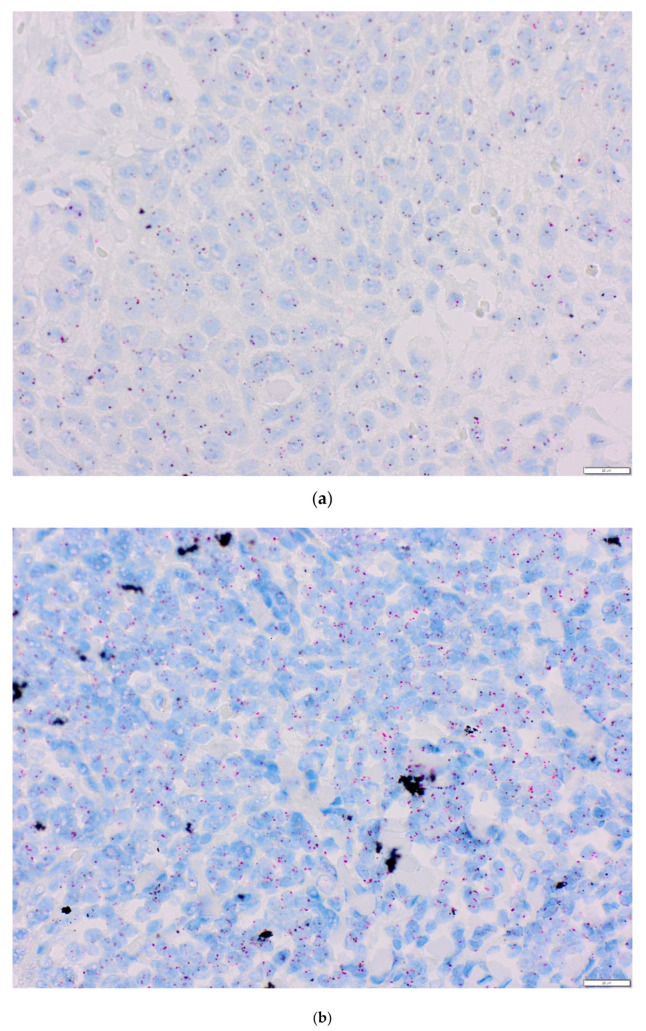
Representative examples of chromogenic in situ hybridization/silver in situ hybridization (CISH/SISH) analysis (x40). Black signals: *PIK3CA*, red signals: chromosome 3 centromere; (**a**) no amplification *PIK3CA/CEP3* < 2, (**b**) amplification *PIK3CA/CEP3* ≥ 2.

In chromogenic (CISH, antigenic labelled probes) and silver (SISH, metallographic detection) in situ hybridization, DNA or RNA fragments are combined with genetic material contained in the test sample with a sequence complementary to the probe. CISH probe was used for centromere 3 (CEP3 DIG Probe) detection with ultraView Red ISH DIG Detection Kit (Ventana Medical Systems, Tuscon, AZ, USA). Silver ion detection in SISH procedure was performed to visualize *PIK3CA* gene (PIK3CA DNP Probe) using ultraView SISH DNP Detection Kit. Both procedures were performed automatically on the BenchMark GX platform (Ventana Roche, Santa Clara, CA, United States). The TMAs were deparaffinated for 4 min at 78 °C. DNA probes were denatured for 20 min at 80 °C and hybridized with PIK3CA DNP Probe and Chromosome 3 DIG Probe at 44 °C for 6 h. After hybridization, three washes of slides at 72 °C were conducted. The results of precipitation of both probes were deposited in the nuclei, and single copies of the *PIK3CA* gene and *CEP3* gene were visualized as a separate discrete dot. The slides were stained by a hematoxylin II solution. CISH red color reaction and SISH black color reaction were evaluated under an optical microscope (Figure 4a–c).

The CISH and SISH methods were used in 89 probes. Three patients were not included due to TMA core loss. The analyses were performed on 50 non-overlapping OSCC cell nuclei. Copies of the *PIK3CA* gene, the number of chromosome 3 DNA centromeres (CEP3), and the ratio (R) of the copy number of the PIK3CA to CEP3 gene were counted. The *PIK3CA* gene copy number and PIK3CA/CEP3 ratio were assessed as a continuous variable. A given case was considered to have amplification when the PIK3CA/CEP3 ratio was above two or for a minimum of 15 copies of the *PIK3CA* gene in the cell nucleus [55,93]. 

### 4.5. Statistical Analysis

Statistical analysis was carried out using the STATISTICA 13.3 (StatSoft Inc., Tulsa, OK, USA) licensed by the Medical University of Gdańsk. All tests were considered statistically significant at *p* ≤ 0.05. Normal distribution of the analyzed variables was verified with W Shapiro-Wilk test. In the analysis of relationships between features, the Pearson χ^2^ independence test and Fisher’s exact test were used. Standard procedures used in this type of research were used in the analysis of prognostic factors. The correlations between the PIK3CA and PTEN protein expression, *PIK3CA* gene amplification, and clinical and pathological patient characteristics clinical were analyzed using Spearman’s R ratio (r).

Survival analysis was analyzed with Kaplan–Meier method and compared by log-rank test or F-Cox test. The univariate analysis results were analyzed based on the log-rank and or F-Cox test results. Multifactorial analysis was performed based on the Cox regression model.

## 5. Conclusions

Down-regulation of PTEN and PIK3CA gene activation is frequently observed in neoplasms, including OSCC. Our data suggest that the loss of PTEN protein may act as a prognostic factor for overall survival. The presence of amplification in tumors located in the lower gingiva resulted in an almost six-fold increase in the risk of shortening survival. Loss of PTEN expression and PIK3CA amplification might be useful as prognostic factors. The prognostic significance of these markers can be confirmed by studies on large groups. It is necessary to study new markers (for example including tyrosine-kinases), standardize detection techniques, and implement new therapeutic methods, including targeted therapies. The markers related to PI3K signaling pathway can be the target point for these therapies, but the potential suitability of targeted therapies on PI3K signaling has to be verified in further studies, e.g., characterizing targets and pathways in OSCC versus benign oral mucosa.

## Figures and Tables

**Table 1:** Clinical and pathological data of patients with oral squamous cell carcinoma (OSCC).

Characteristic	All	PIK3CA Protein Expression			PTEN Protein Expression			Amplification		
		Low Level of PIK3CA	High Level of PIK3CA	*p* Value	Loss of PTEN	Expression of PTEN	*p* Value	No Amplification of the *PIK3CA* Gene	Amplification of the *PIK3CA* Gene	*p* Value
N (%)	92 (100)	44 (48.35)	47 (51.65)	-	28 (31.46)	61 (68.54)	-	84 (94.38)	5 (5.62)	-
**Age, n (%)**										
<60	62 (67.39)	19 (43.18)	19 (39.58)	0.726	14 (50.00)	23 (37.70)	0.279	36 (42.86)	2 (40.00)	0.725
≥60	30 (32.61)	25 (56.82)	29 (60.42)		14 (50.00)	38 (62.30)		48 (57.14)	3 (60.00)	
**Gender, n (%)**										
Male	62 (67.39)	33 (75.00)	29 (60.42)	0.136	22 (78.57)	39 (63.93)	0.171	57 (67.86)	3 (60.00)	0.909
Female	30 (32.61)	11 (25.00)	19 (39.58)		6 (21.43)	22 (36.07)		27 (31.14)	2 (40.00)	
**Localization, n (%)**										
Tongue	23 (25.00)	12 (27.28)	11 (22.92)	0.924	7 (25.00)	15 (24.59)	0.753	22 (26.19)	1 (20.00)	0.040
Buccal mucosa	15 (16.30)	7 (15.90)	8 (16.66)		4 (14.28)	11 (18.04)		15 (17.85)	0 (0)	
Floor of mouth	29 (31.52)	13 (29,55)	16 (33.34)		11 (39.28)	17 (27.87)		26 (30.95)	0 (0)	
Upper gingiva	9 (9.79)	1 (2.72)	8 (16.66)		3 (10.71)	5 (8.20)		9 (10.71)	0 (0)	
Lower gingiva	14 (15.22)	9 (20.45)	5 (10.42)		2 (7.14)	12 (19.67)		10 (11.90)	4 (80.00)	
Hard palate	2 (2.17)	1 (2.72)	1 (2.08)		1 (3.57)	1 (1.64)		2 (1.02)	0 (0)	
**Smoking, n (%)**										
Smoking patients	49 (53.26)	24 (54.55)	25 (52.08)	0.423	12 (42.86)	36 (59.02)	0.055	44 (52.38)	3 (60.00)	0.957
No smoking patients	18 (19.57)	7 (15.91)	11 (22.92)		9 (32.14)	9 (14.75)		16 (19.05)	1 (20.00)	
No data	25 (27.17)	13 (29.55)	12 (25.00)		7 (25.00)	16 (22.23)		24 (28.57)	1 (20.00)	
**Alcohol abuse, n (%)**										
Alcoholics	14 (15.22)	8 (18.18)	6 (12.50)	0.329	4 (14.29)	10 (16.39)	0.745	13 (15.48)	0 (0)	0.382
No alcoholics	52 (56.52)	24 (54.55)	28 (58.33)		17 (60.71)	34 (55.74)		47 (55.95)	3 (60.00)	
No data	26 (28.26)	12 (27.27)	14 (29.17)		7 (25.00)	17 (27.87)		24 (28.57)	2 (40.00)	
**Grading according to WHO, n (%)**										
G1	38 (41.30)	18 (40.91)	20 (41.67)	0.561	12 (42.86)	25 (40.98)	0.671	34 (40.48)	1 (20.00)	0.351
G2	43 (46.74)	23 (52.27)	20 (41.67)		14 (50.00)	28 (45.90)		40 (47.62)	3 (60.00)	
G3	11 (11.95)	3 (6.82)	8 (16.67)		2 (7.14)	8 (13.11)		10 (11.90)	1 (20.00)	
**TNM staging system**										
**T—primary tumor, n (%)**										
T1	16 (17.39)	9 (20.45)	10 (20.45)	0.191	3 (10.71)	13 (21.31)	0.045	20 (23.81)	1 (20.00)	1.000
T2a and T2b	24 (26.09)	13 (29.55)	14 (29.55)		7 (25.00)	16 (26.23)		3 (3.57)	0 (0)	
T3	12 (13.04)	4 (9.09)	4 (9.09)		5 (17.86)	7 (11.48)		7 (8.33)	1 (20.00)	
T4a, T4b	19 (20.65)	8 (18.18)	9 (18.18)		9 (32.14)	9 (14.75)		6 (7.14)	0 (0)	
No data	21 (22.83)	10 (22.73)	11 (22.73)		4 (14.29)	16 (26.23)		48 (57.14)	3 (60.00)	
**N—regional lymph nodes, n (%)**										
N0	37 (40.22)	18 (40.91)	19 (39.58)	0.116	10 (35.71)	26 (42.62)	0.263	33 (39.29)	1 (20.00)	0.921
N1	6 (6.52)	4 (9.09)	2 (4.17)		1 (3.57)	5 (8.20)		6 (7.14)	0 (0)	
N2a, N2b, N2c	14 (15.22)	9 (20.45)	5 (10.42)		9 (32.14)	5 (8.20)		13 (15.48)	1 (20.00)	
N3a and T3b	13 (14.13)	1 (2.27)	12 (25.00)		4 (14.29)	9 (14.75)		13 (15.48)	0 (0)	
No data	22 (23.91)	12 (27.27)	10 (20.83)		4 (14.29)	16 (26.23)		19 (22.62)	3 (60.00)	
**M—distant metastases, n (%)**										
M0	6 (6.52)	5 (11.36)	1 (2.08)	0.061	3 (10.71)	3 (4.92)	0.474	6 (7.14)	0 (0)	0.682
M1	0 (0)	0 (0)	0 (0)		0 (0)	0 (0)		0 (0)	0 (0)	
Mx	64 (69.57)	27 (61.36)	37 (77.08)		22 (78.57)	41 (67.21)		59 (70.24)	2 (40.00)	
No data	22 (23.91)	12 (27.27)	10 (20.83)		3 (10.71)	17 (27.87)		19 (22.62)	3 (60.00)	
**Staging by WHO, n (%)**										
I	16 (17.39)	9 (20.45)	7 (14.58)	0.838	3 (10.71)	13 (21.31)	0.250	15 (17.86)	0 (0)	0.793
II	15 (16.30)	7 (15.91)	8 (16.67)		6 (21.43)	8 (13.11)		12 (14.29)	1 (20.00)	
III	13 (14.13)	3 (6.81)	10 (20.83)		4 (14.29)	9 (14.75)		13 (15.48)	0 (0)	
IV	28 (30.43)	15 (34.09)	13 (27.08)		11 (39.29)	16 (26.23)		27 (32.14)	1 (20.00)	
No data	20 (21.74)	10 (22.73)	10 (20.83)		4 (14.29)	15 (24.59)		17 (20.24)	3 (60.00)	
**Treatment, n (%)**										
Surgery	32 (34.78)	16 (36.36)	16 (33.33)	0.776	11 (39.29)	32,79	0.615	29 (34.52)	0 (0)	0.977
Radiotherapy	10 (10.87)	7 (15.91)	3 (6.25)		7 (25.00)	4,92		10 (11.90)	0 (0)	
Chemotherapy	0 (0)	0 (0)	0 (0)		0 (0)	0 (0)		0 (0)	0 (0)	
Surgery and radiotherapy	21 (22.83)	8 (18.18)	13 (27.08)		6 (21.43)	14 (22.95)		19 (22.62)	2 (40.00)	
Surgery and chemotherapy	1 (1.09)	0 (0)	1 (2.08)		0 (0)	1 (1.64)		1 (1.19)	0 (0)	
Radiotherapy and chemotherapy	5 (5.43)	2 (4.55)	3 (6.25)		2 (7.14)	2 (3.28)		5 (5.95)	0 (0)	
Surgery, radiotherapy and chemotherapy	3 (3.26)	1 (2.27)	2 (4.17)		0 (0)	3 (4.92)		3 (3.57)	0 (0)	
Treatment was not started	6 (6.52)	1 (2.27)	5 (10.42)		0 (0)	6 (9.84)		5 (5.95)	1 (20.00)	
No data	14 (15.22)	9 (20.45)	5 (10.42)		2 (7.14)	12 (19.67)		12 (14.29)	2 (40.00)	
**Five-year survival, n (%)**										
Five-year survival	29 (31.52)	15 (34.09)	14 (29.17)	0.557	4 (14.29)	23 (37.70)	0.030	27 (32.14)	0 (0)	0.127
Patients did not survive 5 years	61 (66.30)	27 (61.36)	34 (70.83)		23 (82.14)	37 (60.66)		55 (65.48)	5 (100.00)	
No data	2 (2.17)	2 (4.55)	0 (0)		1 (3.57)	1 (1.64)		2 (2.38)	0 (0)	

**Table 2:** Phosphatidylinositol-4,5-bisphosphate 3-kinase (PIK3CA) and phosphatase and tensin homolog encoded on chromosome 10 (PTEN) protein expression, *PIK3CA* gene copy number, and amplification versus clinical and pathological patient characteristics (r—correlation coefficient).

Clinical and Pathological Data	PIK3CA Protein Expression		PTEN Expression		*PIK3CA* Gene Copy Number		*PIK3CA/CEP3 Ratio*	
	*p*-Value	*r*	*p*-Value	*r*	*p*-Value	*r*	*p*-Value	*r*
Age [years]	0.766	0.032	0.447	0.082	0.489	−0.074	0.175	−0.145
Gender	0.264	−0.118	0.171	−0.146	0.522	0.069	0.586	0.058
Localization *	0.761	−0.032	0.752	0.034	0.297	0.112	0.078	0.188
Smoking	0.304	−0.128	0.053	−0.239	0.758	0.039	0.357	0.117
Alcohol abuse	0.307	−0.128	0.741	0.042	0.769	0.038	0.275	0.140
Grading	0.293	0.111	0.670	0.046	**0.050**	**0.208**	0.070	0.193
T	0.466	0.089	**0.043**	**−0.243**	0.403	−0.103	0.089	−0.209
N	0.147	0.177	0.263	−0.136	0.960	0.006	0.132	−0.186
M	0.052	−0.235	0.470	−0.088	0.542	−0.076	0.420	−0.100
Staging	0.747	−0.039	0.250	−0.139	0.406	−0.102	0.052	−0.235
Treatment **	0.715	0.042	0.615	−0.059	0.196	0.151	0.151	0.167
5-year survival	0.462	−0.079	**0.028**	**0.235**	0.616	0.054	0.416	0.088

* Localization: tongue, floor of the mouth, buccal mucosa, upper gingiva, hard palate, lower gingiva. ** Treatment: surgery, radiotherapy, chemotherapy, surgery and radiotherapy, surgery and chemotherapy, radiotherapy and chemotherapy, surgery, radiotherapy and chemotherapy, treatment was not started, no data. The correlations between the PIK3CA and PTEN protein expression, *PIK3CA* gene amplification and clinical and pathological patient characteristics clinical were analyzed using Spearman’s R ratio.

**Table 3:** Multivariate Cox’s regression analysis of PIK3CA and PTEN protein expression, *PIK3CA* gene copy number and amplification (HR—hazard ratio, *p*–*p*-Value).

Clinical and Pathological Data	PIK3CA Protein—Continuous Variable			PIK3CA Protein—H-score 70 as Cut-off Point			Loss of PTEN			*PIK3CA* Gene Copy Number			*PIK3CA/CEP3* Ratio			*PIK3CA* Gene Amplification		
	HR	95% CI	*p*	HR	95% CI	*p*	HR	95% CI	*p*	HR	95% CI	*p*	HR	95% CI	*p*	HR	95% CI	*p*
**Age [years]**	1.017	−0.009–0.042	0.205	2.709	−0.172–2.165	0.077	1.013	−0.013–0.039	0.330	1.027	−0.162–0.215	0.783	1.195	−0.249–0.605	0.424	1.570	−0.500–1.402	0.381
**Gender**	1.001	−0.003–0.005	0.623	1.160	−0.347–0.645	0.556	0.600	−1.026–0.003	0.058	1.034	−0.156–0.223	0.727	1.202	−0.239–0.607	0.405	1.660	−0.440–1.454	0.326
**Grading**	1.001	−0.003–0.005	0.750	1.070	−0.428–0.564	0.788	**0.565**	**−1.099–(−0.041)**	**0.040**	1.003	−0.189–0.195	0.976	1.120	−0.315–0.542	0.610	1.563	−0.499–1.393	0.382
**Staging**	1.001	−0.004–0.006	0.633	1.171	−0.408–0.723	0.584	0.556	−1.174–0	0.054	0.985	−0.276–0.247	0.912	1.165	−0.530–0.836	0.659	1.071	−1.397–1.534	0.928
Localisation *	1.001	−0.004–0.006	0.759	1.108	−0.474–0.679	0.727	0.680	−1.026–0.257	0.249	1.092	−0.162–0.340	0.487	1.407	−0.228–0.910	0.244	**5.783**	**0.054–3.456**	**0.037**
**Smoking**	1.003	−0.001–0.002	0.155	1.491	−0.182–0.981	0.175	**0.436**	**−0.148–(−0.177)**	**0.016**	0.960	−0.277–0.195	0.733	1.088	−0.469–0.637	0.768	1.595	−0.597–1.531	0.418
**Alcohol abuse**	1.002	−0.002–0.007	0.449	1.282	−0.346–0.843	0.410	**0.526**	**−1.243–(−0.040)**	**0.042**	0.938	−0.315–0.187	0.614	1.030	−0.562–0.621	0.923	1.429	−0.864–1.577	0.585
**T**	1.001	−0.002–0.006	0.766	1.067	−0.529–0.658	0.831	0.616	−1.096–0.128	0.125	0.976	−0.252–0.203	0.836	1.032	−0.529–0.592	0.913	0.483	−2.314–0.859	0.337
**N**	1.001	−0.003–0.006	0.557	1.351	−0.299–0.900	0.325	0.532	−1.267–0.002	0.054	0.952	−0.310–0.211	0.711	1.018	−0.668–0.704	0.958	0.676	−1.940–1.157	0.604
**M**	1.001	−0.003–0.006	0.582	1.145	−0.434–0.704	0.640	**0.527**	**−1.215–(−0.066)**	**0.033**	1.000	−0.241–0.2413	0.999	1.139	−0.503–0.762	0.686	1.300	−1.195–1.719	0.734
**Surgery**	1.000	−0.004–0.004	0.905	1.135	−0.403–0.656	0.639	**0.539**	**−1.158–(−0.078)**	**0.029**	1.071	−0.104–0.242	0.445	1.215	−0.179–0.568	0.324	1.151	−1.060–1.342	0.822
**Radiotherapy**	1.002	−0.003–0.006	0.470	1.170	−0.397–0.771	0.577	**0.491**	**−1.296–(−0.128)**	**0.020**	0.972	−0.257–0.291	0.809	1.061	−0.470–0.589	0.827	1.331	−0.912–1.484	0.653
**Chemotherapy**	1.002	−0.003–0.006	0.424	1.211	−0.364–0.748	0.498	**0.523**	**−1.127–(−0.079)**	**0.029**	0.946	−0.241–0.181	0.643	1.0	−0.520–0.571	0.928	1.296	−0.948–1.467	0.685

* Localisation: tongue, floor of the mouth, buccal mucosa, upper gingiva, hard palate, lower gingiva.

## Abbreviations

aCGH	array comparative genomic hybridization
AKT	serine-threonine kinase
CISH	chromogenic in situ hybridization
cMET	tyrosine-protein kinase Met
EGFR	epidermal growth factor receptor
FISH	fluorescent in situ hybridization
H-score	hybrid scoring system method
HNSCC	head and neck squamous cell carcinoma
HR	hazard ratio
IHC	immunohistochemistry
MLPA	multiplex ligation-dependent probe amplification
OSCC	oral squamous cell carcinoma
p	p value
PCR	polymerase chain reaction
PDK1	3-phosphoinositide-dependent kinase-1
PH	pleckstrin homology
PI3K	intracellular phosphatidylinositol 3-kinase signaling pathway
PIK3CA or p110	phosphatidylinositol-4,5-bisphosphate 3-kinase
PIP2	phosphatidylinositol 4,5-bisphosphate
PIP3	phosphatidylinositol 3,4,5-trisphosphate
PTEN	phosphatase and tensin homolog encoded on chromosome 10
RT-PCR	reverse transcription polymerase chain reaction
RKT	receptor tyrosine kinases
SISH	silver in situ hybridization
TGFβ/SMAD pathway	transforming growth factor beta/SMAD
TMA	tissue microarray
